# Nuclear autoantigenic sperm protein facilitates glioblastoma progression and radioresistance by regulating the ANXA2/STAT3 axis

**DOI:** 10.1111/cns.14709

**Published:** 2024-04-11

**Authors:** Yuning Qiu, Dongling Pei, Minkai Wang, Qimeng Wang, Wenchao Duan, Li Wang, Kehan Liu, Yu Guo, Lin Luo, Zhixuan Guo, Fangzhan Guan, Zilong Wang, Aoqi Xing, Zhongyi Liu, Zeyu Ma, Guozhong Jiang, Dongming Yan, Xianzhi Liu, Zhenyu Zhang, Weiwei Wang

**Affiliations:** ^1^ Department of Neurosurgery The First Affiliated Hospital of Zhengzhou University Zhengzhou Henan China; ^2^ Academy of Medical Sciences Zhengzhou University Zhengzhou Henan China; ^3^ Department of Pathology The First Affiliated Hospital of Zhengzhou University Zhengzhou Henan China

**Keywords:** annexin A2, glioblastoma, nuclear autoantigenic sperm protein, radioresistance, WP1066

## Abstract

**Aims:**

Although radiotherapy is a core treatment modality for various human cancers, including glioblastoma multiforme (GBM), its clinical effects are often limited by radioresistance. The specific molecular mechanisms underlying radioresistance are largely unknown, and the reduction of radioresistance is an unresolved challenge in GBM research.

**Methods:**

We analyzed and verified the expression of nuclear autoantigenic sperm protein (NASP) in gliomas and its relationship with patient prognosis. We also explored the function of NASP in GBM cell lines. We performed further mechanistic experiments to investigate the mechanisms by which NASP facilitates GBM progression and radioresistance. An intracranial mouse model was used to verify the effectiveness of combination therapy.

**Results:**

NASP was highly expressed in gliomas, and its expression was negatively correlated with the prognosis of glioma. Functionally, NASP facilitated GBM cell proliferation, migration, invasion, and radioresistance. Mechanistically, NASP interacted directly with annexin A2 (ANXA2) and promoted its nuclear localization, which may have been mediated by phospho‐annexin A2 (Tyr23). The NASP/ANXA2 axis was involved in DNA damage repair after radiotherapy, which explains the radioresistance of GBM cells that highly express NASP. NASP overexpression significantly activated the signal transducer and activator of transcription 3 (STAT3) signaling pathway. The combination of WP1066 (a STAT3 pathway inhibitor) and radiotherapy significantly inhibited GBM growth in vitro and in vivo.

**Conclusion:**

Our findings indicate that NASP may serve as a potential biomarker of GBM radioresistance and has important implications for improving clinical radiotherapy.

## Graphical abstract

Nuclear autoantigenic sperm protein (NASP) plays a crucial role in glioblastoma progression and radioresistance, promotes DNA repair, and activates the STAT3 signaling pathway through ANXA2. The NASP/ANXA2/STAT3 axis is a potential therapeutic target for improving the prognosis of patients with GBM.

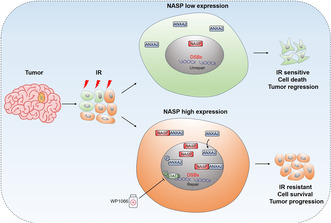

## 1 INTRODUCTION

Gliomas, which are neuroepithelial tumors, are the most common malignant tumors of the central nervous system in adults. Gliomas are broadly categorized into low‐grade gliomas (LGGs) and glioblastoma multiforme (GBM) according to the 2021 World Health Organization (WHO) Central Nervous System Tumor Classification (Fifth Edition).^1^ Currently, surgery followed by postoperative radiotherapy and temozolomide chemotherapy is the first choice of treatment for high‐grade gliomas. However, despite the availability of standardized comprehensive treatment regimens, long‐term therapeutic outcomes remain poor, especially for GBM. For instance, the median survival time of patients with GBM is only 15–17 months, and the 5‐year relative survival rate is only 6.9% even in patients receiving simultaneous radiotherapy and chemotherapy after tumor removal surgery.^2^, ^3^ These findings highlight the importance of elucidating the molecular mechanisms underlying GBM to identify novel potential therapeutic targets.

In addition to surgery, radiotherapy is one of the most important treatment options for patients with GBM.^4^ However, approximately 80% of the patients show relapse after high‐dose radiotherapy owing to progressive radioresistance.^5^, ^6^ Therefore, prevention or reversal of radioresistance may substantially improve GBM outcomes. The radioresistance of GBM is partially mediated by the DNA damage response (DDR), and small‐molecule inhibitors of DDR components (such as poly ADP‐ribose polymerase, ataxia‐telangiectasia mutated kinase, and Wee1) have demonstrated strong radiosensitization efficacy in preclinical models and early clinical trials.^7^, ^8^, ^9^, ^10^, ^11^, ^12^ Therefore, identifying methods to improve the radiosensitivity of GBM is critical for improving patient prognosis.

Nuclear autoantigenic sperm protein (NASP) is a histone molecular chaperone required for DNA replication, cell proliferation, and cell cycle progression. Its main functions include transporting histones into the nucleus of mitotic cells and regulating histone modifications. NASP shows two splice variants: somatic and testicular. The former is mainly expressed in embryonic tissues and somatic cells, whereas the latter is primarily expressed in the testis, stem cells, embryonic tissues, and tumor cells.^13^, ^14^, ^15^, ^16^ Previous studies have shown that downregulation of NASP can inhibit the expression of cell cycle‐related proteins and thereby inhibit the proliferation of renal cancer cells. Conversely, increased NASP expression has been shown to promote melanoma cell proliferation by accelerating the G1/S phase transition of the cell cycle, and high levels of NASP are predictive of shorter overall survival and higher recurrence rates in patients with melanoma.^15^, ^17^, ^18^ Recent studies have also shown that NASP is a target gene of methyltransferase‐like 3 (MELLT3) in GBM and is strongly associated with chemotherapeutic drug resistance.^19^ However, the role of NASP in GBM radioresistance has not been examined.

In the current studies, we investigated the expression level of NASP in glioma (LGG and GBM) and its relationship with patient prognosis. Next, we explored the effect of NASP on the proliferation, migration, invasion, and radioresistance of GBM cell lines. In addition, we demonstrated the molecular mechanism by which NASP mediates DNA repair through ANXA2 in GBM, and observed the effect of combination treatment with the STAT3 pathway inhibitor, WP1066, and radiotherapy in tumor‐bearing mice models. Our results suggest that the NASP/ANXA2/STAT3 axis plays an important role in the malignant progression and radioresistance of GBM, and thus may be a promising therapeutic target for GBM.

## 2 MATERIALS AND METHODS

### 2.1 Patient sample collection

The patient population (totally 232 patients) was selected from patients who underwent surgical resection in 2019 at the Department of Neurosurgery, the First Affiliated Hospital of Zhengzhou University, Zhengzhou, P.R. China. Inclusion and exclusion criteria are described in Supplementary S1. The clinical data of 232 patients with glioma are shown in Table S1.

### 2.2 Cell culture and regents

The GBM cell lines U87 and U251 were obtained from the American Type Culture Collection. Cells were cultured in Dulbecco's modified Eagle's Medium (DMEM, Sigma) supplemented with 10% fetal bovine serum (FBS, Gibco) at 37°C under a humidified 5% CO_2_ atmosphere. All cell lines were tested for mycoplasma every 3 months, and all cells used for the experiments were passaged ≤10 times. The STAT3 inhibitor WP1066 was obtained from MCE (HY‐15312) and administered from stocks prepared in dimethyl sulfoxide and stored at −80°C.

### 2.3 Lentiviral plasmids and lentivirus transduction

U87 and U251 cells were infected with lentiviruses to obtain stable cell lines. Detailed information regarding this procedure is described in Supplementary S2.

### 2.4 Irradiation

Cells were irradiated using an X‐RAD 225 system (Precision X‐ray) at approximately 2 Gy/min. Briefly, the cell culture dishes were placed approximately 50 cm below the radiation probe at different times to deliver the desired dose. For orthotopic tumor irradiation, the animals were anesthetized and positioned such that the apex of each tumor was at the center of the aperture in the secondary collimator, with the remaining mice shielded from radiation.

### 2.5 Cell viability assay

Cell viability was measured using the Cell Counting Kit 8 (CCK8, MCE) according to the manufacturer's instructions. Briefly, cells were seeded on 96‐well plates with 100 μL of culture medium at 5 × 10^3^ cells/well. At 0, 24, 48, 72, and 96 h, 10 μL of CCK‐8 reagent was added to each well for 2 h. Absorbance was measured at 450 nm using a microplate reader (PERLONG, China) to estimate the number of live cells. Some wells were filled with cell‐free medium as a blank control, and the absorbance was subtracted from that of the seeded wells. All experiments were performed in triplicate with independently treated cultures.

### 2.6 EdU staining assay

Cells were cultured in 24‐well plates, treated with 100 μL of medium containing 20 μM EdU at 37°C under a 5% CO_2_ atmosphere for 2 h, fixed with 4% paraformaldehyde for 30 min, and incubated with phosphate‐buffered saline (PBS) containing 0.5% Triton X‐100 for 20 min. The nuclei were counterstained with Hoechst 33342 (Beyotime, China). The proliferation rate was calculated according to the manufacturer's instructions (BeyoClick™ EdU Cell Proliferation Kit with Alexa Fluor 555; Beyotime, China). Three randomly selected regions from each group were imaged using a fluorescence microscope (Leica, Wetzlar, Germany).

### 2.7 Clonogenic survival

Cells in the exponential growth phase were treated with radiation, WP1066, or both and then replated at cloning densities. Cells were grown for 14 days, fixed with 4% paraformaldehyde for 30 min, stained with crystal violet for 2 h, and scored according to the number of colonies with ≥50 cells. Radiation survival data were corrected for plating efficiency by normalizing the values to those obtained for unirradiated control cultures. Cell survival curves were fitted using a linear quadratic equation, and the mean inactivation dose was calculated to determine the radiation enhancement ratio as an indicator of radiation sensitization (ratio > 1) or resistance (ratio < 1).

### 2.8 Wound‐healing assay

Cells were seeded in 6‐well plates at 1 × 10^6^ cells/well and grown overnight to form monolayers. A 200‐μL sterile plastic tip was used to create a wound line (cell‐free area) across the culture plate surface, and deplated cells were removed by washing with PBS. Cells were cultured in serum‐free DMEM under a humidified 5% CO_2_ atmosphere at 37°C for 48 h, and images of the wound lines were acquired using a phase‐contrast microscope (Zeiss, Germany). Each assay was performed in triplicate. The cell migration ability was estimated by measuring the scratch width.

### 2.9 Transwell migration and invasion assays

After serum starvation for 24 h, U87 and U251 cells from different transfection groups were harvested and reseeded at 10^5^ cells/200 μL serum‐free DMEM in the upper chambers of Transwell chambers (Corning, U.S.), while 600 μL of DMEM with 20% FBS was added to the lower chambers as a cellular attractant. After 24 h, non‐migrating cells on the filter side of the upper chamber were removed using a cotton swab, and the polycarbonate membrane of the Transwell chamber was fixed with 4% paraformaldehyde for 30 min, rinsed three times with PBS, and stained with crystal violet for 2 h. For the invasion assay, the Transwell chambers were pre‐coated with Matrigel before cell seeding. Migrating or invading cells were counted under a fluorescence microscope (Zeiss, Germany). All assays were repeated at least thrice using independently treated cultures.

### 2.10 Immunofluorescent staining of γ‐H2AX, NASP, annexin A2, and phospho‐annexin A2 (Tyr23)

Cells were treated with 4% paraformaldehyde. Proteins were immunolabeled with different antibodies for immunofluorescence staining. Detailed information is provided in Supplementary S3.

### 2.11 Co‐immunoprecipitation assay

GBM cell lines overexpressing NASP were seeded on 10‐cm dishes, homogenized in lysis buffer (16H17B08; BOSTER, China) supplemented with a protease inhibitor cocktail (CW2200S; CWBIO, China), and immunoprecipitated with rabbit NASP polyclonal (p)Ab (11323‐1‐AP; Proteintech, China, 1:100), annexin A2 mouse monoclonal (m)Ab (66035‐1‐Ig; Proteintech, 1:100), rabbit IgG isotype control (AC005; ABclonal, China), or mouse IgG isotype control (AC011; ABclonal) at 4°C overnight. The protein mixtures were then incubated with protein A/G beads for 3 h at 4°C. The beads were washed, centrifuged five times with PBS containing a protease inhibitor cocktail at 4°C, resolved with 10% SDS buffer, and analyzed by western blotting.

### 2.12 Immunoprecipitation and mass spectrometry

U87 cells overexpressing NASP were seeded on 10‐cm dishes, lysed, and immunoprecipitated with anti‐IgG or anti‐NASP antibodies. Successful immunoprecipitation of NASP was verified using western blotting. The immunoprecipitated protein was analyzed using liquid chromatography–tandem mass spectroscopy (LC–MS/MS) by Jingjie PTM Biolabs (Hangzhou, China). Detailed information is provided in Supplementary S4.

### 2.13 Nuclear and cytoplasmic protein extraction

Nuclear and cytoplasmic proteins were extracted from the treated cells using a nuclear and cytoplasmic protein extraction kit (Beyotime, China) according to the manufacturer's instructions. Detailed information is provided in Supplementary S5.

### 2.14 Comet assay

Comet assays were performed to detect double‐stranded breaks (DSBs) in DNA using A DNA Damage Detection Kit (KeyGEN Biotech, China). The detailed information is provided in Supplementary S6.

### 2.15 Immunohistochemistry assay

Immunohistochemical staining was performed using a commercial kit (BOSTER, China), Briefly, tumor tissue slices were dewaxed with ethanol and xylene, incubated in citrate buffer for antigen repair, blocked with BSA for 1 h, and then incubated with rabbit NASP pAb (11323‐1‐AP; Proteintech, 1:100), mouse annexin A2 mAb (66035‐1‐Ig; Proteintech, 1:5000), rabbit phospho‐annexin A2 (Tyr23) pAb (AF7096; Affinity, USA, 1:100), rabbit STAT3 pAb (10253‐2‐AP; Proteintech, 1:200), and rabbit phospho‐STAT3 (Y705) pAb (EP2147Y; Abcam, UK, 1:100) at 4°C overnight. The slices were then washed three times with PBS, incubated with anti‐rabbit or anti‐mouse secondary antibodies, washed three times with PBS, and stained with DAB solution (Service Bio, China) under radiotherapy for 5–10 min. Images were captured using a light microscope (Nikon, Tokyo, Japan).

### 2.16 RNA sequencing analysis

RNA sequencing analysis was performed using Novogene Bioinformatics Technology (Beijing, China); detailed information is provided in Supplementary S7.

### 2.17 Reverse transcription‐quantitative real‐time polymerase chain reaction assay

Total RNA was extracted using Trizol (ZOMANBIO, China), and 1000 ng of RNA was reverse‐transcribed into cDNA using the PrimeScript™ RT Reagent Kit with gDNA Eraser (Takara Bio Inc, Japan). Reverse transcription‐quantitative real‐time polymerase chain reaction (RT‐qPCR) was performed using the TaqPro Universal SYBR qPCR Master Mix (Vazyme, China) and the Applied Biosystems 7500 Real‐time PCR System (Thermo Fisher Scientific, USA). Gene expression values were normalized to GADPH, which was used as an internal control. The primer sequences used in this study are listed in Table S2.

### 2.18 Western blotting assay

Cells were lysed in ice‐cold radioimmunoprecipitation assay (RIPA) buffer (CWBio, China) containing protease inhibitors, and the total protein content was quantified using the BCA Protein Assay Kit (Vazyme, China). The lysates were then mixed with the loading buffer and heated to 100°C for 10 min to denature the proteins. Cellular proteins were separated by sodium dodecylsulfate–polyacrilamide gel electrophoresis (SDS‐PAGE), transferred to polyvinylidene difluoride membranes (Millipore, USA), and reacted with rabbit NASP pAb (11323‐1‐AP, Proteintech, 1:1000), mouse annexin A2 mAb (66035‐1‐Ig, Proteintech, 1:5000), rabbit phospho‐annexin A2 (Tyr23) pAb (AF7096, Affinity, USA, 1:1000), rabbit STAT3 pAb (10253‐2‐AP, Proteintech, 1:2000), rabbit phospho‐STAT3 (Y705) pAb (EP2147Y, abcam, UK, 1:2000), and rabbit phospho‐histone H2A.X (Ser139) Ab (# 2577S, Cell Signaling Technology, USA, 1:1000). The blotted membranes were incubated with horseradish peroxidase (HRP)‐conjugated anti‐rabbit IgG/anti‐mouse IgG (1:5000; Proteintech). Finally, immunolabeling was detected using an enhanced chemiluminescence kit (NCM Biotech, China), and band intensity was analyzed from the gel images using ImageJ software.

### 2.19 Intracranial mouse model

A mouse GBM model was established by the intracranial injection of U87 cells, as described by Pierce et al.^20^ Detailed information is provided in Supplementary S8.

### 2.20 Statistical analysis

All datasets are presented as the mean ± standard deviation (SD) with individual data points. Statistical analyses were performed using SPSS (version 19.0; SPSS, Chicago, IL, USA). The Shapiro–Wilk normality test was used to assess data distribution. Datasets showing a normal distribution were compared using Student's *t*‐test, as indicated, while those that did not show a normal distribution were compared using the indicated nonparametric test. Differences between multiple groups were analyzed using one‐way or two‐way ANOVA. We used the log‐rank test in univariate survival analyses, and a Kaplan–Meier plot was used for presentation. Individual *p*‐values are indicated in the figure legends, and a *p*‐value of <0.05 was considered statistically significant for all tests. All experiments were performed independently at least three times.

## 3 RESULTS

### 3.1 NASP is highly expressed in LGG and GBM and is associated with a poor prognosis

**FIGURE 1:**
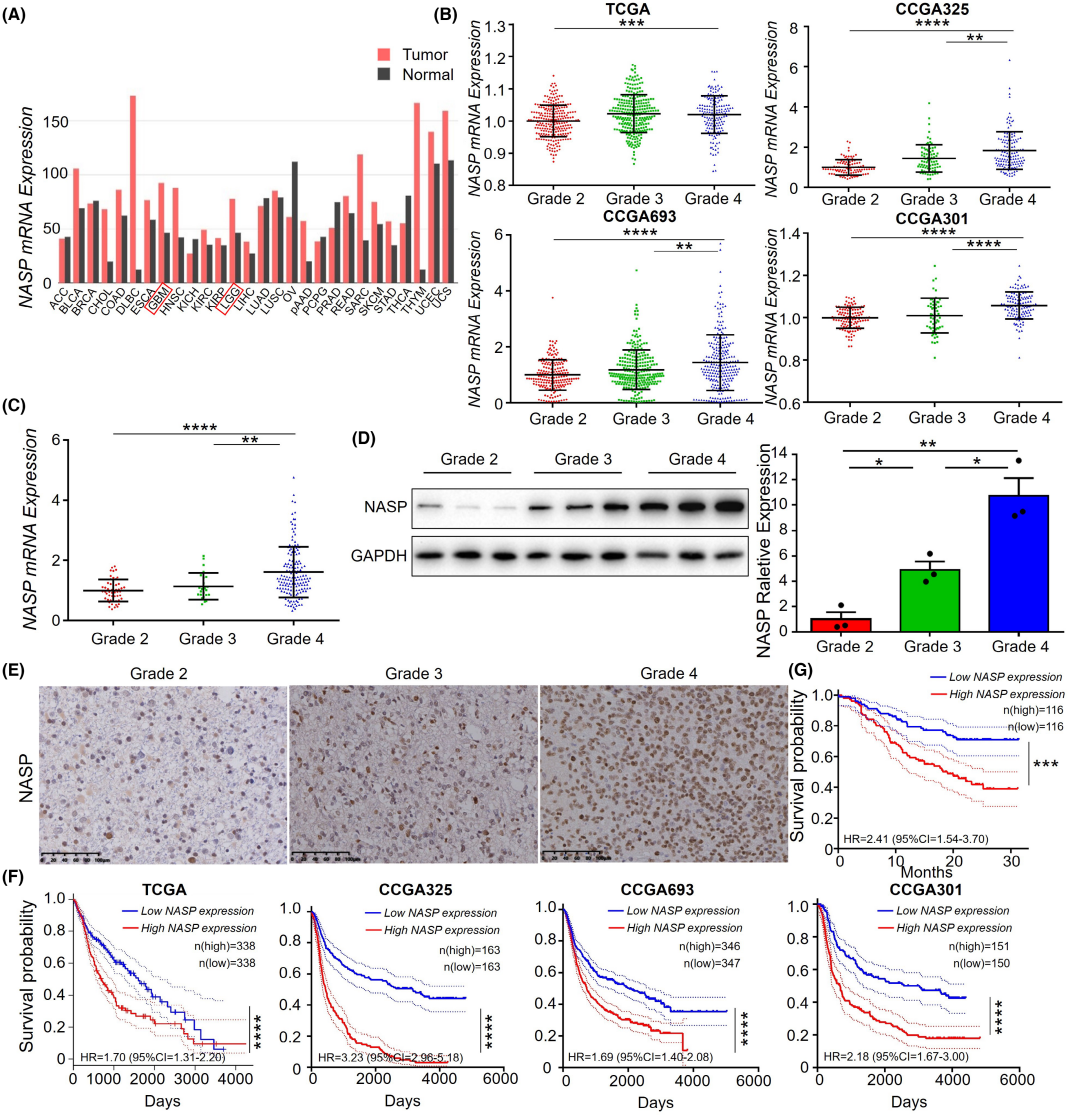
NASP is highly expressed in LGG and GBM and predicts poor prognosis. (A) Expression levels of NASP mRNA in different tumor types and the corresponding normal tissues obtained from the TCGA database. (B) Expression levels of NASP mRNA in glioma tissues of different grades obtained from TCGA and CGGA databases. (C) Levels of NASP mRNA expression in glioma tissues of different grades obtained from the sequencing data of an institutional tumor bank (*n* = 232 samples). (D) Expression of NASP protein in glioma tissues of different grades determined using western blotting. (E) Immunohistochemical staining of NASP protein in glioma tissues of different grades. Scale bars, 100 μm. (F) Kaplan–Meier curves of patient survival in relation to the NASP mRNA expression levels obtained from TCGA and CGGA databases. (G) Kaplan–Meier curves of patient survival in relation to the NASP mRNA expression levels obtained from the sequencing data of an institutional tumor bank. *p < 0.05, **p < 0.01, ***p < 0.001, and ****p < 0.0001 by Student's *t*‐test.

To investigate the role of NASP in glioma development, we first compared the expression of NASP across different types of tumor tissues and the corresponding normal tissues by using The Cancer Genome Atlas (TCGA) database. The results showed that the expression levels of NASP were higher in various tumor tissues, including LGG and GBM, than in the corresponding normal tissues (Figure 1a), suggesting that NASP plays a role in the malignant progression of glioma. Next, we examined the association between NASP expression and glioma grade and found that NASP mRNA expression levels were higher in grade 4 than in grade 2 or 3 gliomas in both the TCGA and Chinese Glioma Genome Atlas (CGGA) cohorts (Figure 1b). To further confirm this association, we performed RNA sequencing of 232 glioma samples of different grades collected at our hospital. As expected, NASP mRNA expression levels were the highest in the grade 4 glioma samples (Figure 1c).

To explore whether the protein expression levels of NASP in gliomas were consistent with their mRNA expression levels, we randomly selected nine glioma tissues of different grades and extracted the total protein content of these tissues. Western blot analysis revealed that the protein expression levels of NASP were positively correlated with the WHO glioma grade. Immunohistochemical analysis of tumor tissues obtained from patients with glioma confirmed this finding (Figure 1d,e).

To explore the effects of NASP expression on the survival time of patients with glioma, we analyzed the survival data obtained from the TCGA and CGGA databases (Figure 1f) and the data of 232 patients with glioma (Figure 1g) collected in our hospital. The results showed that patients with high NASP expression had worse survival rates than those with low NASP expression. The NASP expression level was higher in glioma tissues, and both mRNA and protein expression levels of NASP were proportional to the WHO grade of glioma. Moreover, high expression of NASP was associated with a poor prognosis, further proving that NASP may play an important role in the malignant progression of gliomas.

### 3.2 NASP promotes proliferation, migration, and invasion of GBM cell lines

**FIGURE 2:**
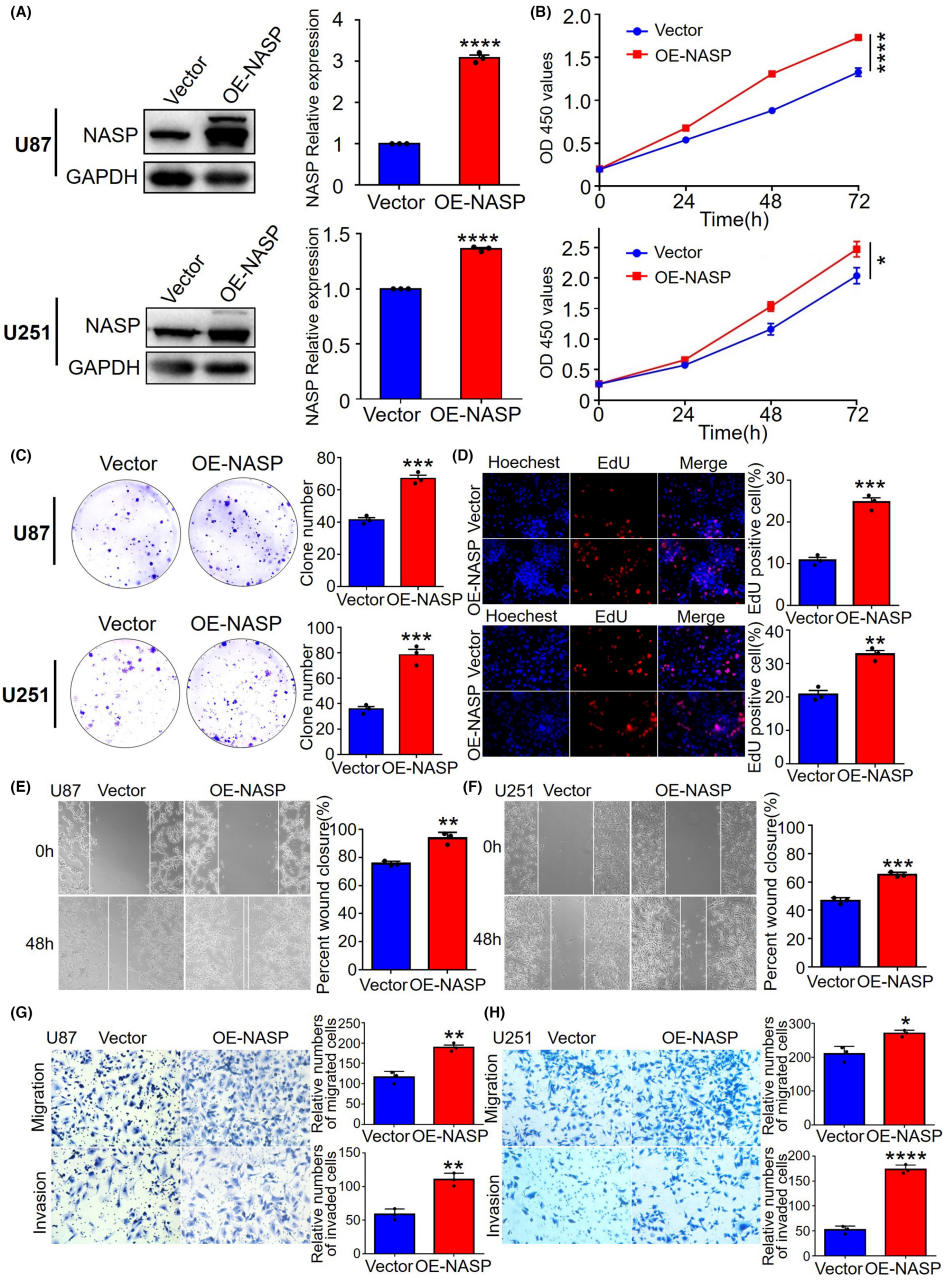
NASP promotes the proliferation, migration, and invasion of GBM cell lines. (A) Western blot assays verified the overexpression efficiency of NASP protein in U87 and U251 cells. (B) CCK8 assays of NASP‐overexpressing and control cell proliferation rates. The upper panel represents U87 cells, and the lower panel represents U251 cells. (C) Clonogenic abilities of NASP‐overexpressing and control U87 and U251 cells. (D) EdU assay to detect the proliferation rates of NASP‐overexpressing and control GBM cells. The upper panel represents U87 cells, and the lower panel represents U251 cells. (E, F) Wound‐healing assay to detect the migration ability of NASP‐overexpressing and control U87 and U251 cells. (G, H) Transwell assays to detect the migration and invasion abilities of NASP‐overexpressing and control U87 (G) and U251 (H) cells. Data are presented as mean ± SD from at least three independent experiments. *p < 0.05, **p < 0.01, ***p < 0.001, and ****p < 0.0001 by Student's *t*‐test.

To determine the biological functions of NASP, we established U87 and U251 cell lines that stably overexpressed and knocked down NASP, respectively, and verified their efficiency using western blotting analysis (Figure 2a and Figure S1a). To explore the effect of NASP on GBM cell proliferation, we performed the CCK8 (Figure 2b), clonogenic (Figure 2c), and EdU proliferation assays (Figure 2d) in U87 and U251 cells stably overexpressing NASP. The results showed that NASP upregulation significantly increased U87 and U251 cell proliferation, whereas the cell lines with NASP knocked down exhibited opposite results (Figure S1b,c). Next, we determined the effects of NASP on GBM cell migration and invasion using wound‐healing assays. We found that NASP upregulation promoted GBM cell migration (Figure 2e,f), whereas NASP downregulation inhibited this effect (Figure S1d). Furthermore, Transwell assays confirmed that the upregulation of NASP promoted the migratory and invasive abilities of GBM cells (Figure 2g,h). These results demonstrate that NASP promotes the proliferation, migration, and invasion of GBM cells, which partly explains the poor prognosis of patients with gliomas showing high NASP expression.

### 3.3 NASP enhances the radioresistance of GBM cells by increasing DNA repair ability

**FIGURE 3:**
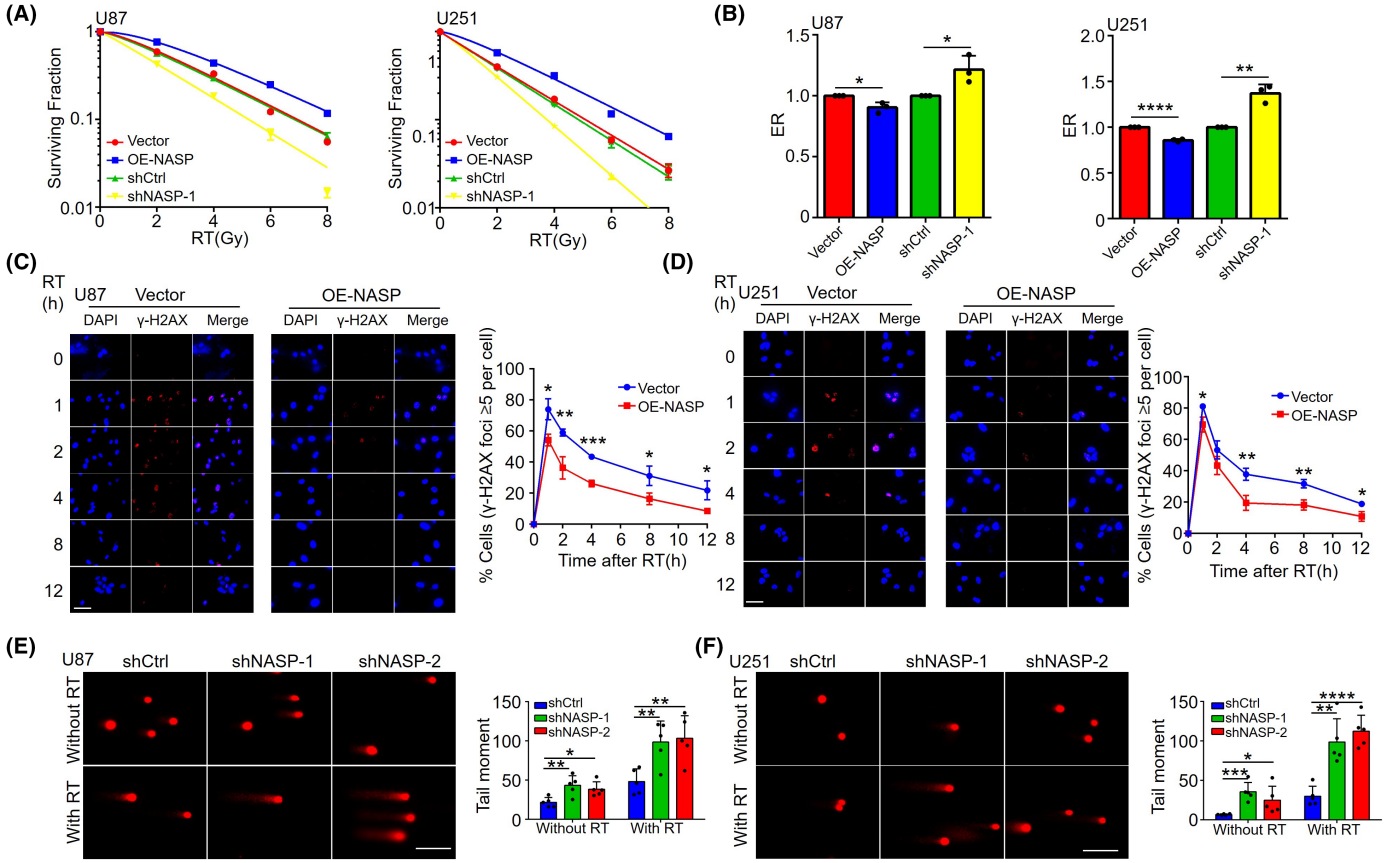
Overexpression of NASP increases the radioresistance of GBM cells by promoting DNA repair. (A, B) Radiotherapy (0, 2, 4, 6, or 8 Gy) was performed on U87 and U251 cell lines overexpressing NASP or with downregulated NASP, and colony formation assays were performed to calculate the survival fraction (A) and radiation enhancement ratio (B). (C, D) DNA double‐strand breaks detected on the basis of γ‐H2AX levels in radiotherapy‐treated and untreated NASP‐overexpressing U87 (C) and U251 (D) cells. Left panels show images of γ‐H2AX foci in cells at various time points. Scale bars, 50 μM. Right panels present the statistics. (E, F) DNA double‐strand breaks detected by comet assays in radiotherapy‐treated and untreated NASP‐knockdown U87 (E) and U251 (F) cells (4 Gy). The left panel shows fluorescence images of comet assays. Scale bars, 100 μM. Right panel shows the statistics. Data are presented as mean ± SD from at least three independent experiments. *p < 0.05, **p < 0.01, ***p < 0.001, and ****p < 0.0001 by Student's *t*‐test.

Human NASP is involved in many cellular processes, including histone transport, cell cycle, cell proliferation, and stem cell proliferation.^21^ Alekseev et al. reported that NASP binds to H1 histones and influences cell cycle progression by mediating DNA–H1 histone binding,^22^ while Richardson et al. reported that NASP is involved in chromatin assembly after DNA replication.^23^ NASP also binds to KU70/KU80 and DNA‐PK in HeLa cells, suggesting that it may be involved in DNA repair,^24^ which in turn confers radioresistance. Therefore, we used a clonogenic assay to examine whether NASP affects the radioresistance of GBM cells. In comparison with the control group, NASP upregulation induced radioresistance in both U87 and U251 cells, whereas NASP downregulation induced radiosensitivity (Figure 3a,b).

To explore the mechanisms underlying NASP‐induced radioresistance, we focused on the role of NASP in DDR. Previous studies have shown that the G2/M checkpoint pathway is upregulated during DNA damage to prevent entry into the M phase and allow DNA repair.^25^ Next, we performed RT‐qPCR to detect the expression levels of key genes involved in DNA repair and the G2/M checkpoint pathway. The results showed that NASP overexpression in U87 and U251 cells significantly upregulated the expression of genes such as *ZWINT*, *FEN1*, *ADRM1*, and *TYMS* (Figure S2a,b).

On the basis of these results, we also investigated the role of NASP in radiotherapy‐induced DNA repair. Immunofluorescence analysis of γ‐H2AX levels revealed that in comparison with control cells, NASP‐overexpressing U87 cells showed significantly reduced DNA damage and a significantly higher rate of reduction of γ‐H2AX levels (Figure 3c). Thus, the DNA repair ability of NASP‐overexpressing cells was higher than that of control cells. Similar experiments were performed using the U251 cell line, which revealed similar results (Figure 3d). For NASP knockdown U87 and U251 cells, we performed comet assays and found that NASP knockdown increased DNA damage in both radiotherapy‐treated and untreated cells in comparison with the control cells (Figure 3e,f). Collectively, these results suggest that NASP plays an important role in DNA repair in GBM cells and is responsible for radioresistance in NASP‐overexpressing GBM cells.

### 3.4 NASP participates in ANXA2‐mediated DNA repair

**FIGURE 4:**
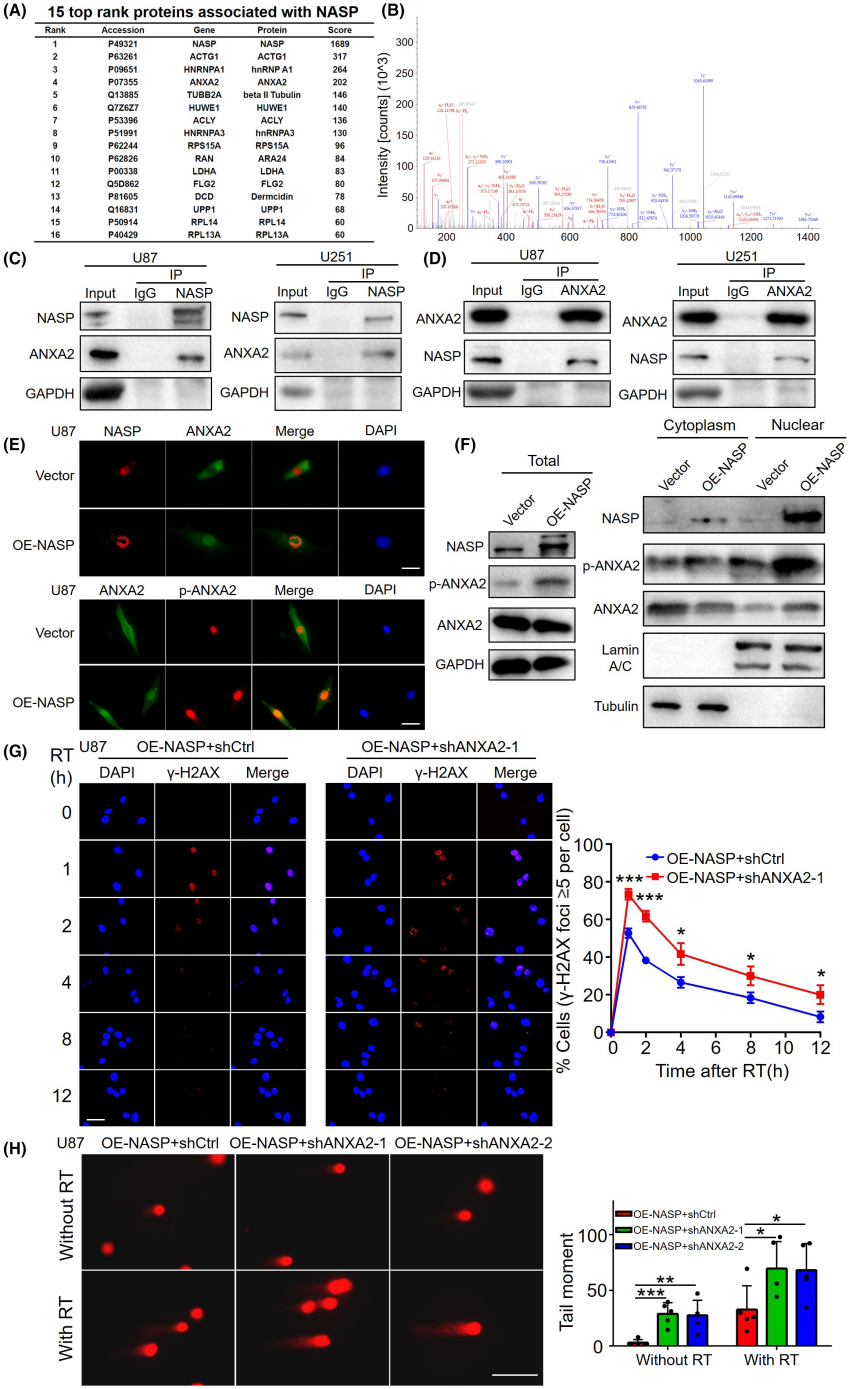
NASP promotes ANXA2‐mediated DNA repair. (A) The top 15 proteins potentially associated with NASP were isolated by immunoprecipitation and identified by mass spectrometry in NASP‐overexpressing U87 cells. Cell lysates were immunoprecipitated with an antibody against NASP, while an antibody against IgG was used as the negative control. (B) Mass spectrogram of ANXA2. (C, D) Western blot detection of NASP and ANXA2 proteins by reciprocal immunoprecipitation with an antibody against NASP (C) or ANXA2 (D) in U87 and U251 cells. IgG was used as the control. (E) Immunofluorescence detection of NASP, ANXA2, and p‐ANXA2 localization in control and NASP‐overexpressing U87 cells. Scale bars, 50 μM. (F) Abundance of NASP, ANXA2, and p‐ANXA2 in cytoplasmic, nuclear, and total protein fractions from the control and NASP‐overexpressing U87 cells estimated by western blotting. (G) Effects of NASP upregulation and ANXA2 downregulation on DNA double‐strand breaks in U87 cells after radiotherapy as detected by immunofluorescence. Scale bars, 50 μM. (H) Comet assay showing the effect of NASP upregulation and ANXA2 downregulation on DNA double‐strand breaks in U87 cells after RT. Scale bars, 100 μM. Data are presented as mean ± SD from at least three independent experiments. *p < 0.05, **p < 0.01, and ***p < 0.001 by Student's *t*‐test.

To investigate how NASP promotes DNA repair, we first identified the potential interacting proteins by performing immunoprecipitation assays on NASP‐overexpressing U87 cell lysates, followed by mass spectrometry analysis. The results showed that at least 44 proteins may interact with NASP (Table S3). Protein scoring according to protein abundance revealed that ANXA2 (ranked No. 3) was a strong candidate (Figure 4a), and the mass spectrogram confirmed the presence of ANXA2 protein in the immunoprecipitates (Figure 4b). ANXA2 is an abundant cellular protein mainly localized to the cytoplasm and membrane, but is also found in small amounts in the nucleus.^26^ Madureira et al. reported that both radiation and genotoxic substances, such as etoposide and hexavalent chromium, cause ANXA2 to accumulate in the nucleus and protect DNA from damage.^27^ Therefore, we speculate that NASP may promote ANXA2‐mediated DNA repair after radiotherapy. To test this hypothesis, we first performed co‐IP assays using lysates of U87 and U251 cells. These experiments revealed ANXA2 protein in lysates treated with the anti‐NASP antibody (Figure 4c) and NASP protein in lysates treated with the anti‐ANXA2 antibody (Figure 4d). These findings further confirmed the interaction between NASP and ANXA2 in GBM cells.

Previous studies have shown that annexin 2 is translocated to the nucleus and that the localization of ANXA2 may depend on tyrosine 23 phosphorylation.^27^, ^28^ Therefore, we examined whether the interaction between NASP and ANXA2 influences the phosphorylation state and nuclear localization of ANXA2. Immunofluorescence assays showed that NASP was mainly distributed in the nucleus of control U87 cells, whereas ANXA2 was mainly distributed in the cytoplasm. However, in NASP‐overexpressing U87 cells, nuclear ANXA2 expression was significantly higher, whereas its cytoplasmic localization was relatively reduced. Moreover, immunostaining revealed that NASP and ANXA2 were colocalized in the nucleus. NASP overexpression also increased the nuclear localization of p‐ANXA2 (Figure 4e). To further verify this co‐localization, we extracted the cytoplasmic, nuclear, and total proteins from U87 cells and conducted separate western blot assays. Consistent with the immunofluorescence results, NASP overexpression enhanced ANXA2 abundance in the nuclear protein fraction, reduced it in the cytoplasmic fraction, and did not alter the total protein expression. NASP overexpression also increased p‐ANXA2 levels in the nuclear and cytoplasmic protein fraction, and increased total expression (Figure 4f). We performed similar experiments on U251 cells, which revealed similar results (Figure S3a). These results suggest that the interaction between NASP and ANXA2 increases the nuclear localization of ANXA2, which may be achieved by increasing tyrosine 23 phosphorylation.

Next, to determine whether DNA repair was mediated through the interaction between NASP and ANXA2, we knocked down ANXA2 expression in NASP‐overexpressing cells. We found that downregulation of ANXA2 reversed the reduction of γ‐H2AX foci caused by overexpression of NASP (Figure 4g), and reversed the reduction in the tail moments of DNA comets caused by NASP overexpression (Figure 4h). These results suggest that NASP participates in ANXA2‐mediated DNA repair by increasing ANXA2 nuclear localization and that its upregulation increases DNA repair ability after radiotherapy.

### 3.5 NASP overexpression excessively upregulates STAT3 signaling

**FIGURE 5:**
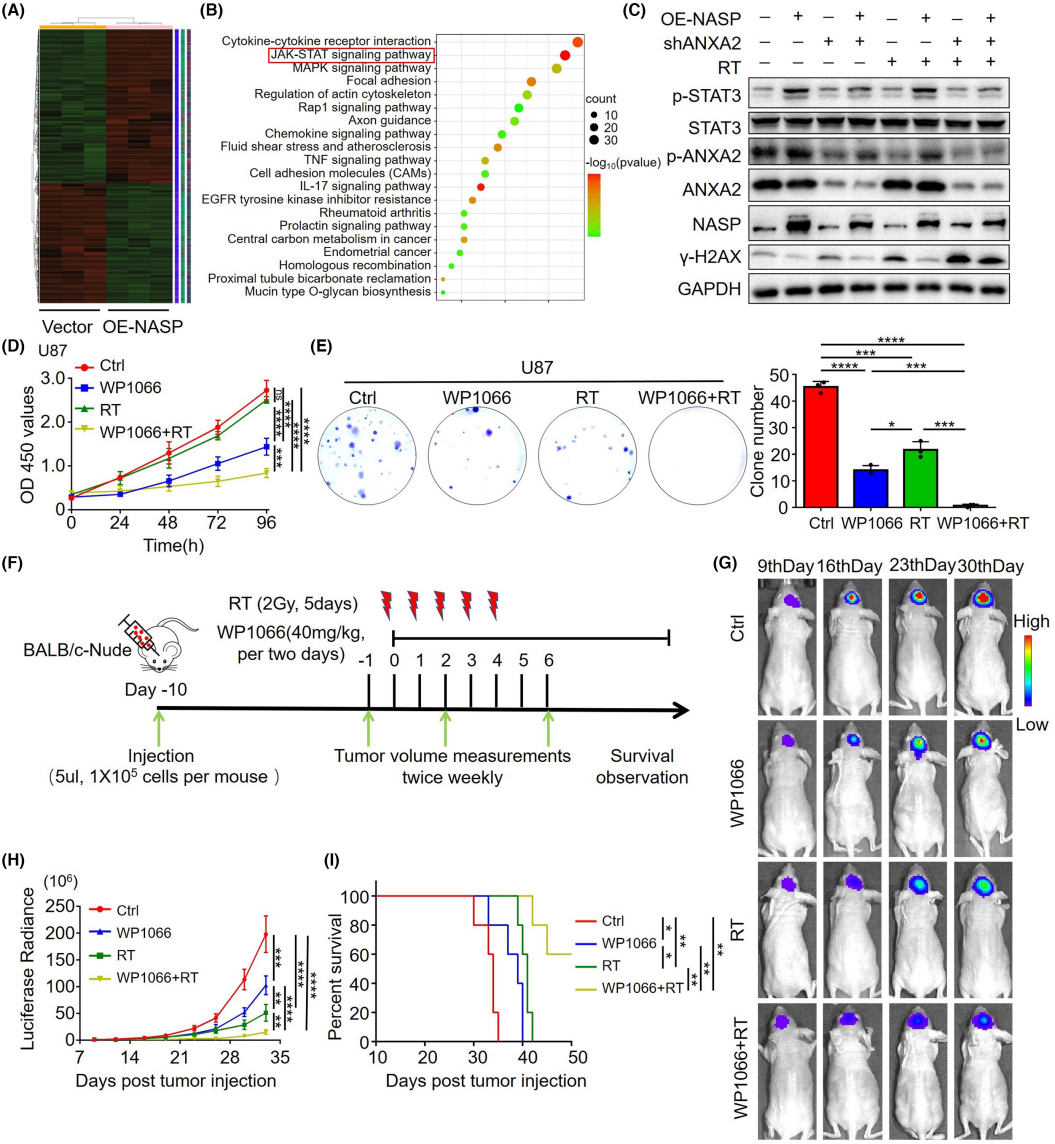
NASP overexpression excessively upregulates STAT3 signaling. (A) Heatmap showing gene expression differences between control and NASP‐overexpressing U87 cells. (B) KEGG pathway enrichment analysis of genes differentially expressed by NASP‐overexpressing U87 cells. (C) STAT3, p‐STAT3, NASP, ANXA2, p‐ANXA2, and γ‐H2AX protein levels in U87 cells (treated as indicated) estimated using western blot analyses. (D, E) Combined application of the STAT3 inhibitor WP1066 and radiotherapy significantly inhibited the proliferation of U87 cells. Effects of WP1066 (10 μM) or WP1066 plus radiotherapy on cell proliferation, as measured using the CCK‐8 assay (D) and colony formation assay (E). Data are presented as mean ± SD from at least three independent experiments. (F) Workflow of the BALB/c‐nude mouse orthotopic tumor model. U87 cells (1 × 10^5^) were injected into the brain of the mouse, and radiotherapy was started on the 10th day (5 doses of 2 Gy) with or without intragastric administration of WP1066 every 2 days (*n* = 5 mice per group). (G, H) In vivo bioluminescence images showing the implanted tumors in the mice treated with WP1066 and (or) RT. (I) Kaplan–Meier curves showing the percentage survival of mice implanted with U87 cells and treated with WP1066 and (or) RT. *p < 0.05, **p < 0.01, ***p < 0.001, and ****p < 0.0001.

Although we proved that NASP promotes ANXA2‐mediated DNA repair, no inhibitors of NASP or ANXA2 are currently available. To further translate our findings into clinical practice and improve the prognosis of patients with GBM, we performed RNA sequencing of NASP‐overexpressing and control U87 cells to identify the pathways involved. Differential gene expression analysis revealed that 1137 and 881 genes were upregulated and downregulated, respectively, in NASP‐overexpressing cells in comparison with the control cells (Figure 5a and Table S4). Kyoto Encyclopedia of Genes and Genomes (KEGG) analysis showed that these differentially expressed genes were mainly enriched in cytokine–cytokine receptor interactions, followed by the JAK/STAT signaling pathway (Figure 5b). Furthermore, western blotting revealed that NASP overexpression increased the phosphorylation level of STAT3 Y705 in radiotherapy‐treated and untreated U87 and U251 cells (Figure 5c and Figure S3b). Consistent with our findings, Rocha et al.^29^ reported that ANXA2 overexpression activated the STAT3 pathway in colorectal cancer, and Yuan et al.^30^ reported that phosphorylation of ANXA2‐Tyr23 was key to activating the STAT3 pathway in breast cancer. Therefore, we further aimed to determine the relationship of ANXA2 and p‐ANXA2 with the STAT3 pathway. Western blotting analyses revealed that the level of p‐ANXA2 was positively correlated with that of p‐STAT3, while the level of ANXA2 exhibited no significant correlation with that of p‐STAT3. Consistent with the previously obtained results, NASP expression was negatively correlated with γ‐H2AX levels, and knocking down ANXA2 could, to some extent, reverse the NASP overexpression‐induced γ‐H2AX reduction (Figure 5c and Figure S3b). Overall, these results indicate that the interaction between NASP and ANXA2 not only promotes DNA repair but also increases the phosphorylation of ANXA2‐Tyr23, and an increase in p‐ANXA2 levels may lead to activation of the STAT3 pathway.

Next, we explored whether STAT3 pathway inhibitors could enhance the effects of GBM radiotherapy. WP1066, a STAT3 pathway inhibitor, can penetrate the blood–brain barrier and is currently undergoing phase I clinical trials for adult recurrent glioma, metastatic melanoma, and high‐grade pediatric brain tumors.^31^ In the present study, we tested the inhibitory effects of different concentrations of WP1066 on the STAT3 pathway in GBM cells. The results showed that 10 μM WP1066 could almost completely inhibit phosphorylated STAT3 Y705 levels in U87 and U251 cells (Figure S3c). Subsequently, we treated these cells with WP1066 in combination with radiotherapy and found that this combination significantly inhibited tumor cell proliferation (Figure 5d and Figure S3d). Clonogenic assays also revealed that the inhibitory effect of WP1066 combined with radiotherapy on the clonogenicity of GBM cells was greater than that of the inhibitors or radiotherapy alone (Figure 5e and Figure S3e). Next, we used U87 cells to establish a mouse orthotopic tumor model and subjected it to combination treatment with WP1066 and radiotherapy. Consistent with the above findings, in vivo experiments also revealed that in comparison with the single‐treatment group, the combined‐treatment group exhibited significantly increased tumor inhibition and significantly prolonged survival time (Figure 5f–i and Figure S3f). These results demonstrate that NASP activates the STAT3 pathway and that WP1066, an inhibitor of the STAT3 pathway, enhances the therapeutic effect of radiotherapy on GBM in vitro and in vivo.

### 3.6 NASP expression predicts the vulnerability of LGG and GBM to radiotherapy

**FIGURE 6:**
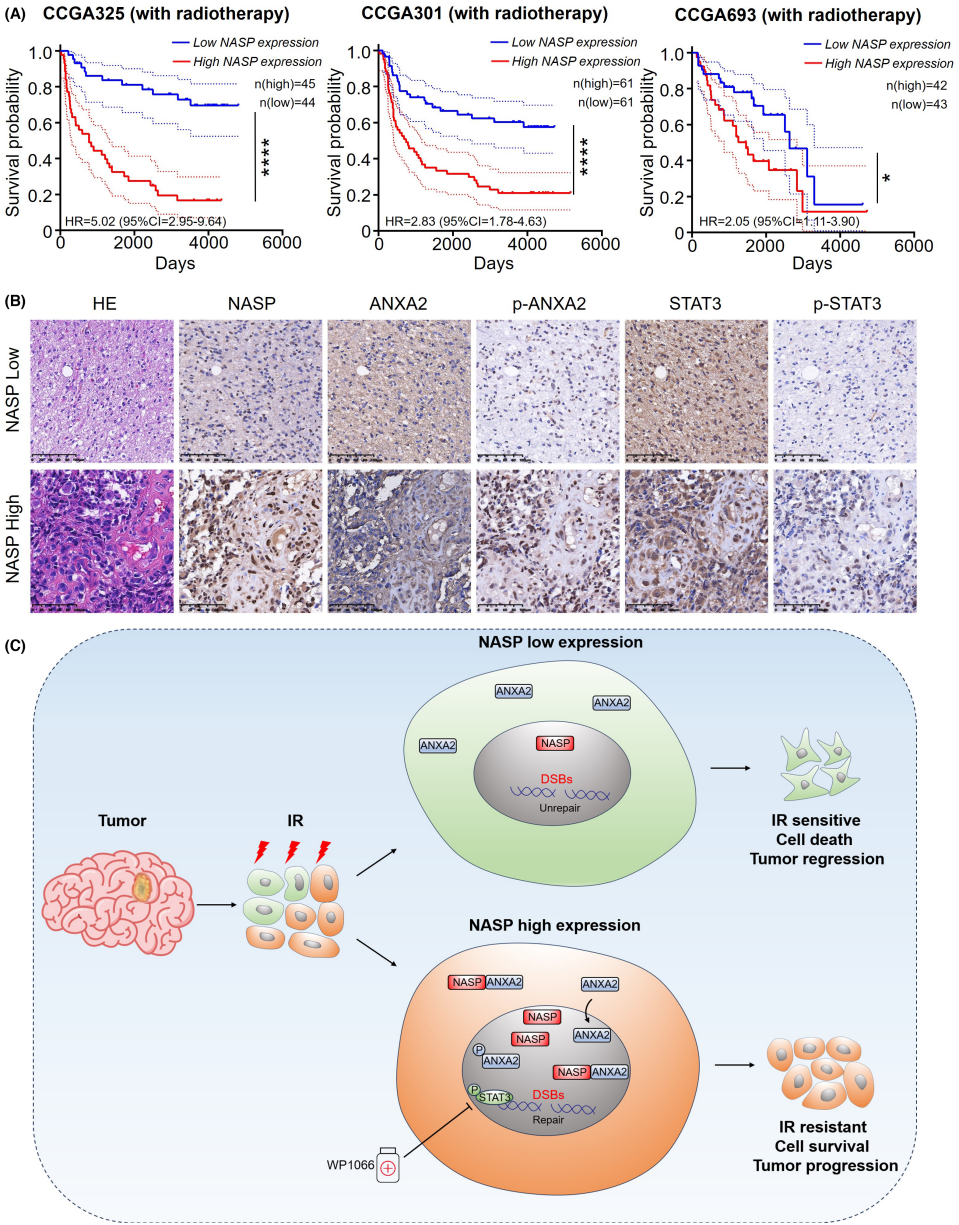
NASP expression predicts the vulnerability of LGG and GBM to radiotherapy. (A) Kaplan–Meier curves of patient survival in relation to NASP mRNA expression levels in glioma patients receiving radiotherapy but not chemotherapy. (B) Immunohistochemical findings for the correlation between NASP expression levels and ANXA2, p‐ANXA2, STAT3, and p‐STAT3 levels in human GBM tissues. Scale bars, 100 μM. (C) Schematic illustration of the possible mechanisms underlying the NASP‐mediated radioresistance of GBM cells. *p < 0.05, **p < 0.01, ***p < 0.001, and ****p < 0.0001.

To determine the influence of NASP expression levels on the radiotherapy response of patients with LGG and GBM, we compared the overall survival rates among patients with glioma in the CGGA database who received radiotherapy but not temozolomide chemotherapy. Consistent with the in vivo findings, high NASP expression was associated with poor overall survival even after radiotherapy (Figure 6a). Consistent with the in vitro experiments, p‐STAT3 and p‐ANXA2 expression levels were higher in tumor tissues with high NASP expression than in those with low NASP expression, whereas no significant difference was observed in ANXA2 and STAT3 expression levels (Figure 6b). In different datasets from the CGGA database, patients with high expression levels of NASP and ANXA2 showed significantly lower survival rates than those with low expression levels of both (Figure S4a–c). Collectively, these results strongly suggest that elevated NASP expression in GBM contributes to poor clinical outcomes by reducing tumor radiosensitivity. Therefore, NASP may be a useful biomarker of GBM response to radiotherapy. Furthermore, blockade of the STAT3 pathway may enhance the response of GBM to radiotherapy, thereby improving the prognosis.

## 4 DISCUSSION

Radiotherapy is the primary treatment option for many types of cancer, including GBM.^32^ Although radiation‐induced DNA damage can lead to tumor cell death, DNA damage, and repair are regulated both intracellularly and extracellularly. In some cases, tumor cells may exhibit radioresistance due to cell‐intrinsic mechanisms and the microenvironment, leading to treatment failure and tumor recurrence.^33^ However, the genetic and molecular mechanisms underlying radioresistance in GBM remain poorly understood, limiting the efficacy of radiotherapy. In this study, we found that NASP expression was correlated with a poor prognosis and promoted GBM radioresistance. Mechanistically, high NASP expression increases its binding to ANXA2 and enhances the nuclear localization of ANXA2 and phosphorylated ANXA2 (Tyr23). This, in turn, enhances the repair of radiotherapy‐induced DNA damage, thereby reducing DSBs and increasing cell survival. Additionally, NASP may activate the STAT3 pathway through p‐ANXA2, which, in turn, promotes radioresistance and tumor progression. In both in vivo and in vitro models, the combination of the STAT3 pathway inhibitor WP1066 and radiotherapy significantly delayed tumor progression (Figure 6c). These findings indicate that the NASP/ANXA2/STAT3 axis may serve as a new target for improving the efficacy of GBM radiotherapy.

In this study, we demonstrated for the first time that NASP promotes the repair of DNA damage in GBM after radiotherapy, thus explaining the radioresistance of GBM. Previous studies have shown that NASP promotes the proliferation of various human cancer cells, including hepatocellular carcinoma, prostate cancer, melanoma, and gastric cancer, and can be used as a marker of poor prognosis.^17^, ^34^, ^35^, ^36^ Our results are consistent with the findings showing that NASP is highly expressed in LGG and GBM and is associated with a poor prognosis. Previous studies have shown that NASP facilitates tumor proliferation and invasion mainly by promoting the G1/S phase transition of the cell cycle^17^, ^18^ or by acting as a target gene of the RNA methyltransferase MELLT3, which is closely related to resistance to various chemotherapy drugs.^19^ However, in this study, we demonstrated that NASP increased the radioresistance of GBM by promoting DNA repair. In a cell model, NASP overexpression promoted the activation of DNA repair pathways and repair of DNA damage, which increased the clonogenic ability of tumor cells after radiotherapy. Therefore, we speculate that GBM with high NASP expression may respond poorly to radiotherapy, which was confirmed by the survival data of patients with glioma after radiotherapy. We propose that NASP can serve as a biomarker of poor prognosis and radioresistance in GBM.

Mechanistically, we demonstrated that NASP promotes DSB repair by interacting with ANXA2. Previous studies have shown that radiation and genotoxic substances such as etoposide and hexavalent chromium lead to increased nuclear localization of ANXA2, which protects DNA from damage.^27^ However, we found that the interaction between NASP and ANXA2 also promoted increased nuclear localization of ANXA2, potentially explaining the reduced DNA damage observed in NASP‐overexpressing cells after radiotherapy. The specific molecular mechanisms underlying ANXA2‐mediated DNA damage repair and the mechanisms by which NASP promotes the transfer of ANXA2 from the cytoplasm to the nucleus remain unclear. Previous studies have shown that the cell‐surface localization of ANXA2 depends on the phosphorylation state of tyrosine 23.^28^ Our study also demonstrated that NASP overexpression increases ANXA2 Tyr23 phosphorylation. Therefore, we speculate that increased phosphorylation of ANXA2 Tyr23 may be responsible for the translocation of ANXA2 in the nucleus, but the specific role of this phenomenon remains to be further explored. Nonetheless, we present strong evidence that the NASP/ANXA2 axis plays a key role in mediating radioresistance in GBM.

Another important result is that the STAT3 pathway was significantly activated in NASP‐overexpressing cells. STAT3 is a transcription factor involved in tumor initiation, progression, malignant behavior, and chemotherapy resistance.^37^, ^38^, ^39^, ^40^ In addition, several studies have shown that STAT3 is involved in the regulation of tumor radioresistance. Luke et al. found that STAT3 inhibition increases radiation‐induced apoptosis.^41^ Additionally, STAT3 is involved in DNA damage repair by regulating BRCA1,^42^, ^43^ and STAT3 inhibition attenuates the efficiency of DNA repair by downregulating the ATM/Chk2 and ATR/Chk1 pathways.^44^ In the current study, the STAT3 pathway was significantly enriched in NASP‐OE cells, and western blotting confirmed that NASP promoted the activation of the STAT3 pathway, which may explain the higher proliferative and invasive abilities and radioresistance of NASP‐OE GBM cells. Consistent with previous studies showing that ANXA2‐Tyr23 phosphorylation is critical for STAT3,^30^ the level of STAT3 Y705 phosphorylation was directly proportional to that of ANXA2 Tyr23 phosphorylation, suggesting that NASP activates the STAT3 signaling pathway through ANXA2‐Tyr23 phosphorylation. While no inhibitors of NASP or ANXA2 are available at present, many inhibitors of the STAT3 pathway have been identified and may be used to improve the efficacy of radiotherapy. We have shown that the STAT3 pathway inhibitor WP1066 increases the radiosensitivity of GBM. Additionally, WP1066 effectively passes through the blood–brain barrier. Phase I clinical trials have been conducted on adult recurrent gliomas, metastatic melanomas, and high‐grade pediatric brain tumors.^31^ Therefore, based on our findings, WP1066 can be used as an effective radiotherapy sensitizer in combination with radiotherapy.

In summary, we demonstrated that NASP promotes tumor progression and radioresistance and provides a plausible molecular mechanism involving ANXA2 phosphorylation and nuclear translocation, as well as enhanced STAT3 signaling, which promotes radioresistance and enhances tumorigenic behavior. We also demonstrated that WP1066, an inhibitor of the STAT3 pathway, enhanced the therapeutic effect of radiotherapy on GBM. Collectively, our findings suggest that the NASP/ANXA2/STAT3 axis is a potential therapeutic target for improving the prognosis of patients with GBM, which has important implications for the development of more effective and precise cancer treatments.

## AUTHOR CONTRIBUTIONS

Weiwei Wang, Zhenyu Zhang, Xianzhi Liu, Yuning Qiu, Dongling Pei designed and directed the research; Dongming Yan, Yuning Qiu, Minkai Wang collected samples; Yuning Qiu, Minkai Wang, Qimeng Wang, Dongling Pei performed the experiments; Wenchao Duan, Li Wang, Kehan Liu, Yu Guo, Lin Luo, Zhixuan Guo, Fangzhan Guan, Zilong Wang, Aoqi Xing, Zhongyi Liu, Zeyu Ma, Guozhong Jiang contributed to writing, discussion and agreement with the conclusions presented. All authors have read and approved the final version of the manuscript.

## CONFLICT OF INTEREST STATEMENT

The authors declare no competing interests.

## Supporting information


Figure S1.

Figure S2.

Figure S3.

Figure S4.

Table S1.

Table S2.

Table S3.

Table S4.

Data S1.

## Data Availability

All data needed to interpret the results are presented in this article and its supplementary information files. Other data that support the findings of our study are available from the corresponding author upon request.
