# Supplementary figures and images for: Nuclear autoantigenic sperm protein facilitates glioblastoma progression and radioresistance by regulating the ANXA2/STAT3 axis
Source: CNS Neurosci Ther. 2024 Apr 11;30(4):e14709. doi: 10.1111/cns.14709 (PMC11009454; doi:10.1111/cns.14709)

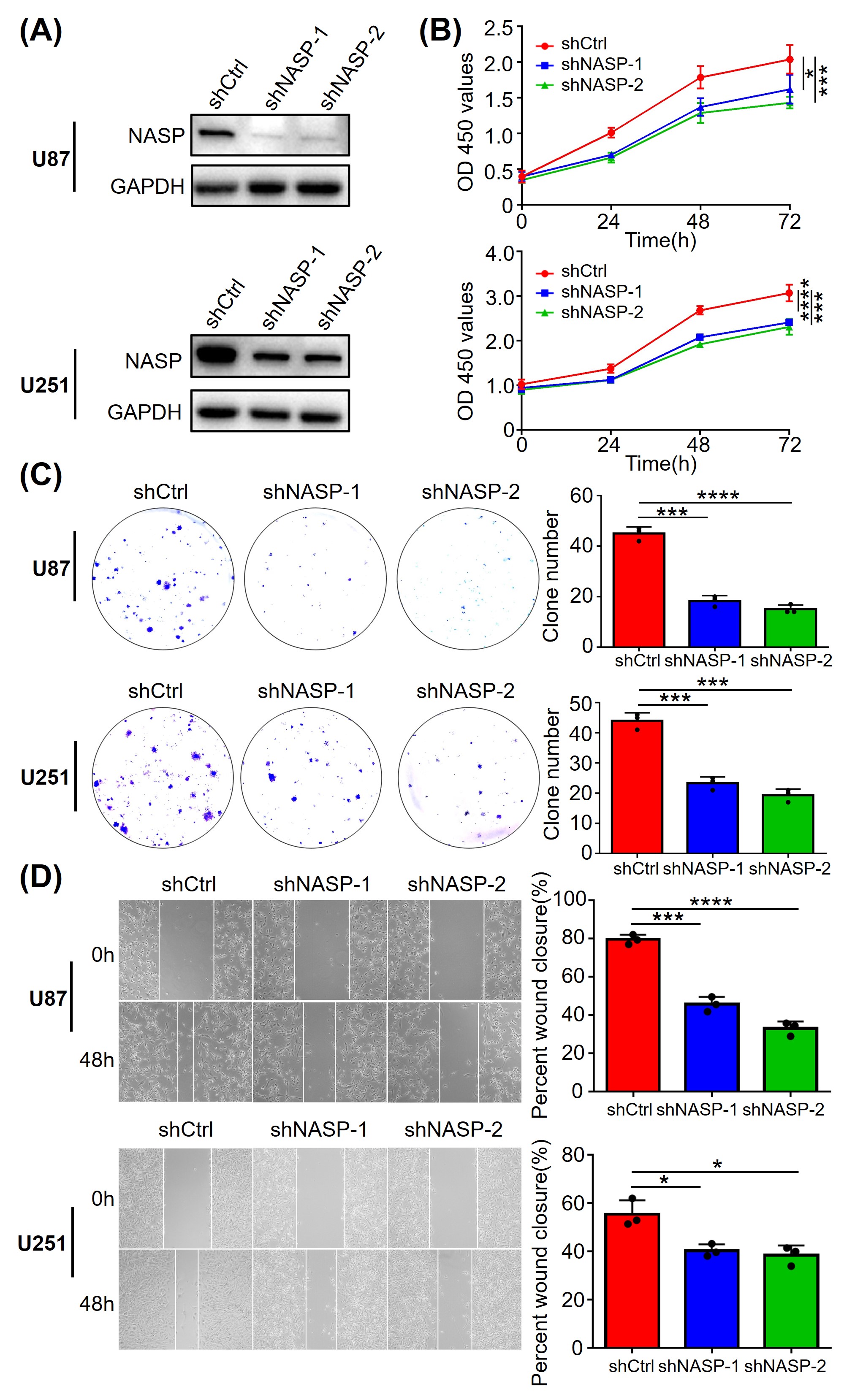

Supplement: Supplementary file 1 — Figure S1. [file CNS-30-e14709-s008.tif]

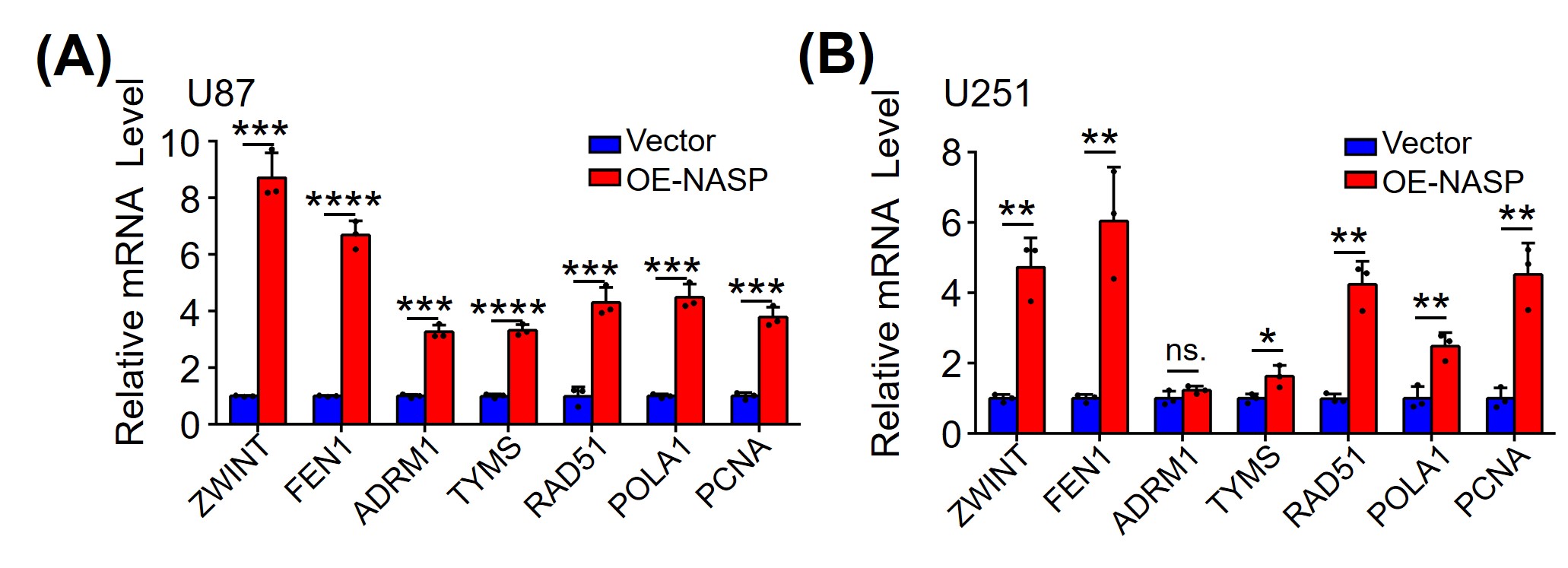

Supplement: Supplementary file 2 — Figure S2. [file CNS-30-e14709-s009.tif]

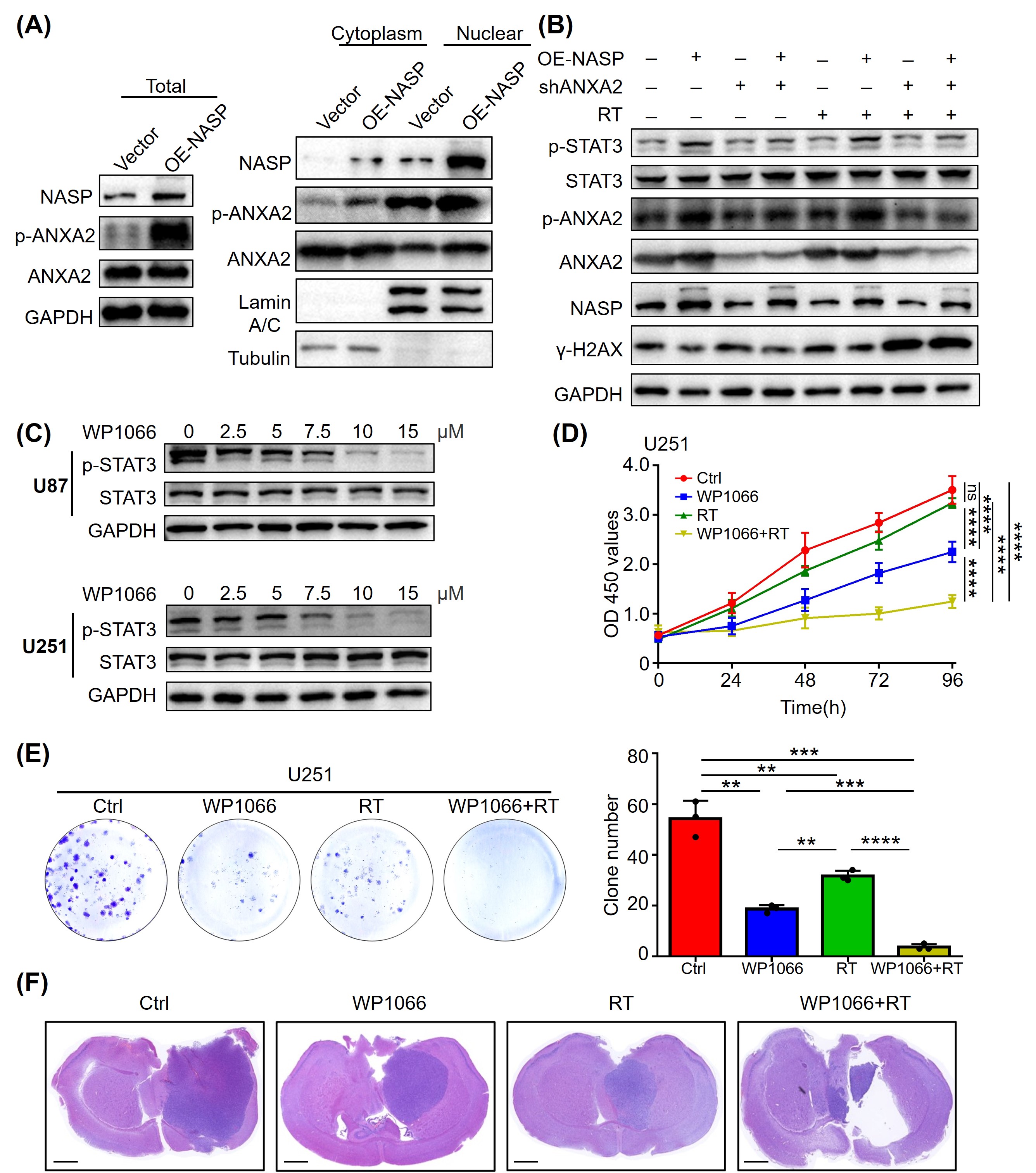

Supplement: Supplementary file 3 — Figure S3. [file CNS-30-e14709-s007.tif]

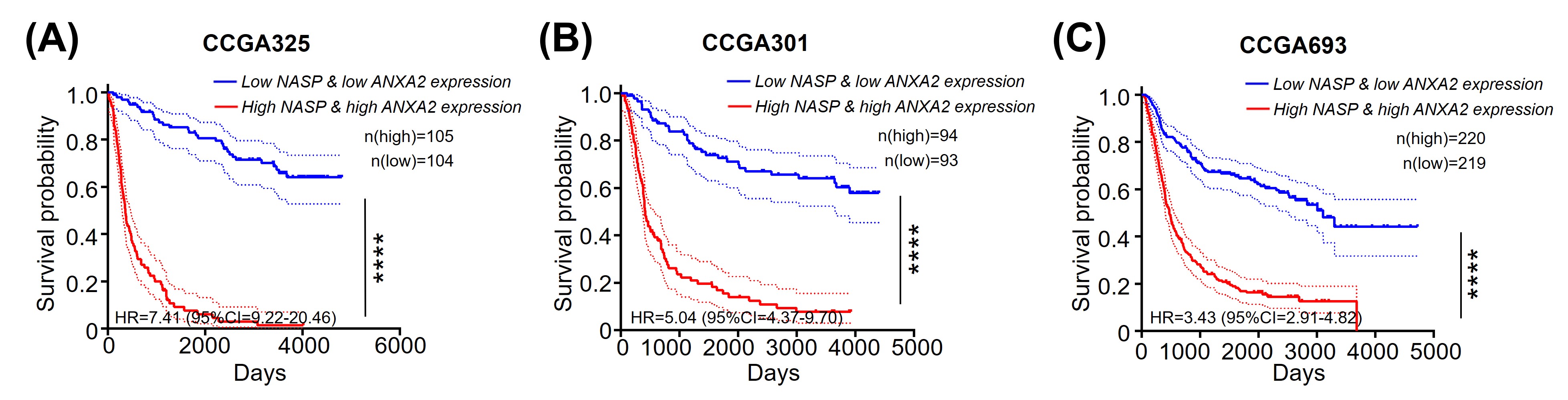

Supplement: Supplementary file 4 — Figure S4. [file CNS-30-e14709-s002.tif]
