# Supplementary material for: Nuclear autoantigenic sperm protein facilitates glioblastoma progression and radioresistance by regulating the ANXA2/STAT3 axis
Source: CNS Neurosci Ther. 2024 Apr 11;30(4):e14709. doi: 10.1111/cns.14709 (PMC11009454; doi:10.1111/cns.14709)
Supplement: Supplementary file 6 — Table S2. [file CNS-30-e14709-s003.docx]

**Table S2 Sequences of primers for RT-qPCR**

| NASP | Forward primer | CTGAAAACGAGGCAGGAAAG |
| --- | --- | --- |
|  | Reward primer | CTGCACCATCCTTAGGGAGA |
| ZWINT | Forward primer | GCATCTTGGAACCTGTAGGC |
|  | Reward primer | GCCTTCAGCTCTTTCCATTG |
| FEN1 | Forward primer | ACATGGACTGCCTCACCTTC |
|  | Reward primer | CCCAATACCCCGGATACTCT |
| ADRM1 | Forward primer | GACGGACGACTCGCTTATTC |
|  | Reward primer | TCCTGGTCTGTCTTGGGTTC |
| TYMS | Forward primer | TCTGGAAGGGTGTTTTGGAG |
|  | Reward primer | CCTCCACTGGAAGCCATAAA |
| RAD51 | Forward primer | TTTGGAGAATTCCGAACTGG |
|  | Reward primer | CATCACTGCCAGAGAGACCA |
| POLA1 | Forward primer | GCCAGCAGAGGAAGTGAAAC |
|  | Reward primer | CCCTTTTACCAATGGGAGGT |
| PCNA | Forward primer | GGCGTGAACCTCACCAGTAT |
|  | Reward primer | TCTCGGCATATACGTGCAAA |
| GAPDH | Forward primer | GGAGTCCACTGGCGTCTTCA |
|  | Reward primer | GTCATGAGTCCTTCCACGATACC |
