# Supplementary material for: Nuclear autoantigenic sperm protein facilitates glioblastoma progression and radioresistance by regulating the ANXA2/STAT3 axis
Source: CNS Neurosci Ther. 2024 Apr 11;30(4):e14709. doi: 10.1111/cns.14709 (PMC11009454; doi:10.1111/cns.14709)
Supplement: Supplementary file 9 — Data S1. [file CNS-30-e14709-s006.docx]

**Supplementary Material**

**This supplementary material includes:**

Supplementary S1: Patient recruitment

Supplementary S2: Lentiviral plasmids and lentivirus transduction

Supplementary S3: Immunofluorescent staining of γ-H2AX, NASP, annexin A2, and phospho-annexin A2 (Tyr23)

Supplementary S4: Immunoprecipitation and mass spectrometry

Supplementary S5: Nuclear and cytoplasmic protein extraction

Supplementary S6: Comet assay

Supplementary S7: RNA sequencing analysis

Supplementary S8: Intracranial mouse model

**Supplementary S1: Patient recruitment**

A total of 232 patients were included in this study. The selection criteria were as follows: (1) glioma diagnosed by neuroimaging and pathology; (2) first-onset and first surgical resection; (3) no preoperative radiotherapy or chemotherapy; and (4) no other nervous system disease. The exclusion criteria were as follows: (1) cerebral hernia or tumor-associated stroke before surgery, (2) severe systemic diseases, (3) mental disorders, or (4) age < 18 years. All enrolled patients were followed up by phone or hospital visits. This study was approved by the College Accreditation Committee of Zhengzhou University and conducted in accordance with the Declaration of Helsinki. Human glioma specimens were used in accordance with the principles of the Ethics Committee of Zhengzhou University.

**Supplementary S2: Lentiviral plasmids and lentivirus transduction**

FUB-MCS-P2A-EGFP-T2A-puro, pLKO.1-TRC-copGFP-T2A-puro, pMD2.G, and psPAX2 plasmids were provided by Dr. Chenglin Zhang (Sino-British Research Centre for Molecular Oncology, Zhengzhou University, China). For gene overexpression, the NASP nucleotide was ligated into the lentivirus plasmid vector FUB-MCS-P2A-EGFP-T2A-puro). For gene knockdown, the shRNA oligonucleotide was ligated into pLKO.1-TRC-copGFP-T2A-puro, followed by lentiviral packaging and infection. The scramble sequence was 5′-CCTAAGGTTAAGTCGCCCTCG-3. ’ The shRNAs targeting NASP sequences were 5′- GCCAGAGGAGAAGGTAGTTAC-3′ (shNASP-1) and 5′- TGCTGCCCAGGCACATCTTAA-3′ (shNASP-2). The shRNAs targeting ANXA2 sequences were 5′- GCAGGAAATTAACAGAGTCTA-3′ (shANXA2-1) and 5′- CGGGATGCTTTGAACATTGAA-3′ (shANXA2-2). All constructs were verified by Sanger sequencing.

To establish GBM cell lines that stably overexpressed or underexpressed the target proteins, U87 and U251 cells were transfected for 72 h with recombinant lentiviruses constructed as described in the preceding section. Virus-infected GBM cells were then selected by continuous culture in 5 μg/mL puromycin. Transfection efficiency was determined by reverse transcription quantitative real-time polymerase chain reaction (RT-qPCR) and western blotting.

**Supplementary S3: Immunofluorescent staining of γ-H2AX, NASP, annexin A2, and phospho-annexin A2 (Tyr23)**

GBM cells were seeded on coverslips at 50%–60% confluence, allowed to attach for 12 h under standard culture conditions, and then exposed to irradiation (4 Gy) or sham treatment (control). At the indicated time points, cells were removed from the radiation source, washed with PBS, fixed in 4% paraformaldehyde for 30 min, washed thrice in PBS, permeabilized with 0.5% Triton X-100, blocked with 1% bovine serum albumin (BSA) in PBS containing 0.1% Triton X-100 for 30 min at room temperature, and incubated with antibodies against phospho-histone H2A.X (Ser139) (# 2577S; Cell Signaling Technology, USA, 1:500), NASP (11323-1-AP; Proteintech, 1:100), annexin A2 (66035-1-Ig; Proteintech, 1:500), and/or phospho-annexin A2 (Tyr24) (AF7096; Affinity, USA, 1:200). The cells were stained with Alexa Fluor 555-labeled donkey anti-rabbit IgG (H+L) secondary antibody (A0453; Beyotime) or Alexa Fluor 488-labeled goat anti-mouse IgG (H+L) secondary antibody (A0428; Beyotime), counterstained with DAPI (D9542-1MG; Sigma, USA), and photographed under a confocal microscope (Leica, Germany).

**Supplementary S4: Immunoprecipitation and mass spectrometry**

Proteins were digested in trypsin, and the tryptic peptides were dissolved in solvent A (0.1% formic acid, 2% acetonitrile/in water), followed by separation on a nanoElute UHPLC system (Bruker Daltonics) with an in-house reversed-phase analytical column (25 cm length, 100 μm i.d.). After exposure to a capillary electrospray source, the charged peptides were subjected to timsTOF Pro mass spectrometry (Bruker Daltonics) in parallel accumulation serial fragmentation mode. The MS/MS data were processed using the MaxQuant search engine (v.1.6.6.0), and the tandem mass spectra were compared against the human SwissProt database (20,366 entries) and the reverse decoy database with a threshold false discovery rate of <1%. The relative amounts of modified peptides in different samples were obtained by centralizing the signal intensity values. After filtering the lysine lactylation sites (localization probability > 0.75), the relative amounts of modified peptides in each sample were obtained from two independent experiments. The ratios of the quantified lysine lactylation peptides were normalized to their corresponding protein expression levels. Biological pathways involving differentially expressed lactylated proteins (DELPs) were identified using the Kyoto Encyclopedia of Genes and Genomes (KEGG) database, and the STRING database was used to identify protein–protein interactions of the DELPs.

**Supplementary S5: Nuclear and cytoplasmic protein extraction**

Cells treated as indicated were washed with PBS, harvested using a cell scraper, mixed with kit reagent A supplemented with PMSF, vortexed for 5 s to disperse the aggregates, and placed on ice for 10–15 min. Reagent B was then added, followed by vortexing for 5 s, incubation on ice for 1 min, and centrifugation at 12000–16000 × *g* for 5 min at 4℃. The supernatant containing the cytoplasmic protein was retained for further analysis, whereas the remaining precipitate was resuspended in a nuclear protein extraction reagent containing PMSF. The mixture was vortexed for 30 s and incubated on ice for 2 min. These steps were repeated 10 times, and the final mixture was centrifuged at 12000–16000 × *g* for 10 min at 4℃. The supernatant was used as the nuclear protein fraction.

**Supplementary S6: Comet assay**

Cells treated as indicated (4 Gy irradiation or control conditions) were collected and suspended in ice-cold PBS for 2 h. The cell density of the suspension was adjusted to 1 × 10^6^/ mL, and a 10-μL sample was mixed with 75 μL of low melting point agarose on a slide precoated with 100 μL of 1% normal melting point agarose. The slides were placed in a precooled lysis buffer for 1 h, washed with PBS, and placed in an electrophoresis tank for 20 min. The slides were incubated three times in Tris–HCL solution (pH 7.5) for 10 min each and treated with 20 μL of propidium iodide for 10 min. Labeled cells were photographed under a microscope, and comet tail moments were analyzed using the Comet Assay Software Project software.

**Supplementary S7: RNA sequencing analysis**

Total RNA was extracted from control and NASP-overexpressing U87 cells using Trizol (Invitrogen, USA) qualified and assessed using a NanoDrop and an Agilent 2100 bioanalyzer (Thermo Fisher Scientific, USA). Magnetic beads with oligo (dT) attachments were used to purify mRNA. The purified mRNA was fragmented into tiny pieces using fragment buffer at an optimum temperature. Later, utilizing random hexamer-primed reverse transcription, first-strand cDNA was synthesized, followed by second-strand cDNA synthesis. A-Tailing Mix and RNA Index Adapters were added and incubated to complete repair. The cDNA fragments derived from the previous step were confirmed by PCR (98°C for 10 seconds, 60°C for 30 seconds, and 72°C for 30 seconds, 15 cycles). The products were purified using Ampure XP Beads before being dissolved in EB solution. The product was confirmed using the Agilent Technologies 2100 bioanalyzer for quality control. To create the final library, the double-stranded PCR products from the previous stage were heated, denatured, and circularized by the splint oligo sequence. The finished library was formatted as single-strand circular DNA (ssCir DNA). The final library was amplified by phi29 to create a DNA nanoball (DNB) containing over 300 copies of one molecule. DNBs were put onto the patterned nanoarray, and single-end 50 base reads were obtained on the BGIseq500 platform (BGI, China). The produced RNA-seq libraries were subjected to gene sequencing.

SOAPnuke (v1.5.2) was used to filter the sequencing data by (1) removing reads that contained a sequencing adapter, (2) removing reads that had a low-quality base proportion (base quality ≤5) of >20%, and (3) removing reads that had an unidentified base (N base) ratio of much more than 5%; clean reads were acquired and saved in FASTQ format. The clean reads were aligned to the reference human genome utilizing HISAT2 (v2.0.4). The clean reads were then matched to the reference coding gene set using Bowtie2 (v2.2.5), and the gene expression level was estimated using RSEM (v1.2.12). Basically, DESeq2(v1.4.5) with a Q value of ≤0.05 was used to perform differential expression analysis. According to the Hypergeometric test, KEGG (https://www.kegg.jp/) enrichment analyses of annotated gene expression among different expressed genes was performed by Phyper (https://en.wikipedia.org/wiki/Hypergeometric_distribution) to gain insight into the change in phenotype. The Q value was adjusted using Bonferroni with a strict threshold (Q value ≤ 0.05) to account for the significant amounts of terms and pathways.

**Supplementary S8: Intracranial mouse model**

Nude mice were anesthetized with isoflurane and fixed in a stereotaxic headframe. The skin was cut to expose the skull, a 1-mm hole was drilled into the anterior fontanelle, and 5 μL of a 2 × 107 cells/mL suspension was slowly injected into the brain with a microinjection needle. Injected cells also stably expressed luciferase for tracing. The scalp was then sutured and the mice were transferred to a heating pad for recovery. Starting 10 days post-inoculation, mice were administered 40 mg/kg WP1066 or DMSO: PEG300 (20:80) (vehicle control) by oral gavage three times per week. Treatment was continued until the study endpoint. Bioluminescent images of tumors were acquired using an IVIS Spectrum imaging system (PerkinElmer).
